# Age- and sex-stratified detection rates and associated factors of colorectal neoplasia in the Tianjin colorectal cancer screening program from 2012 to 2020

**DOI:** 10.1186/s12876-023-03060-3

**Published:** 2023-12-12

**Authors:** Zhen Yuan, Shuyuan Wang, Yuqi Wang, Hongzhou Li, Weifeng Gao, Xinyu Liu, Youkui Han, Zhaoce Liu, Qinghuai Zhang, Hong Ma, Junying Wang, Xiaomeng Wei, Xipeng Zhang, Wei Cui, Chunze Zhang

**Affiliations:** 1 School of Medicine, Nankai University, Tianjin, China; 2 Department of Endoscopy, Tianjin Union Medical Center, Tianjin, China; 3 Department of Colorectal Surgery, Tianjin Union Medical Center, Tianjin, China; 4 Tianjin Medical University, Tianjin, China; 5 Department of General Surgery, Tianjin Union Medical Center, Tianjin, China; 6 The Institute of Translational Medicine, Tianjin Union Medical Center of Nankai University, Tianjin, China; 7 Tianjin Institute of Coloproctology, Tianjin, China; 8 Department of Nursing, Tianjin Union Medical Center, Tianjin, China; 9 Hospital Infection Management Division, Tianjin Union Medical Center, Tianjin, China; 10 School of Mathematical Sciences and LPMC, Nankai University, Tianjin, China

**Keywords:** CRC screening, Cancer prevention, Risk factors, Early-onset CRC

## Abstract

**Purpose:**

Colorectal cancer (CRC) screening has been implemented in Tianjin, China since 2012. The objective was to estimate the neoplasia detection rate in a high-risk population by age and sex and to investigate the potential factors associated with colorectal neoplasia.

**Patients and methods:**

This study is based on data of the Tianjin CRC screening program from 2012 to 2020. Residents with a positive high-risk factors questionnaire (HRFQ) or a positive faecal immunochemical test (FIT) were identified as high-risk participants and were subsequently recommended for a free colonoscopy.

**Results:**

A total of 4,117,897 eligible participants aged 40–74 years completed both a HRFQ and FIT, and 217,164 (5.3%) of them were identified as high-risk participants. Positive rates of preliminary screening increased with age and were higher in females than in males. For 57,971 participants undertaking colonoscopy, the detection rates of nonadvanced adenoma, advanced adenoma and CRC were 37.8%, 5.7% and 1.6%, respectively. Detection rates of advanced neoplasia increased from the age of 50 and were higher in males. For nonadvanced neoplasia, a strong increase was observed in males from the age of 40 and in females from the age of 50. Male sex had a greater impact on individuals aged 40–49 than on older individuals. Several factors including current smoking, drinking, and higher body mass index (BMI) were significantly associated with the presence of neoplasia, whereas, these associations were mainly restricted to individuals aged above 50 but not those aged 40–49 years.

**Conclusions:**

These findings support that age-specific risk stratification and sex-specific initiating ages for CRC screening should be recommended to improve the accuracy and effectiveness of current screening strategy.

**Supplementary Information:**

The online version contains supplementary material available at 10.1186/s12876-023-03060-3.

## Introduction

Colorectal cancer (CRC) is the fifth most common cause of mortality in China [1] and it showed an increasing trend in the incidence and mortality rates over the past decades [2]. In Tianjin, China, CRC has also exhibited a notable disease burden, with an incidence rate of 30.37/100,000 [1]. Most CRCs develop from adenomas, among which advanced adenomas are considered to be the clinically relevant precursors of CRC [3]. The projected annual transition rates from advanced adenoma to CRC are 2.6%-5.6% and increase strongly with age [4]. The slow progression of CRC from a benign neoplasm to invasive carcinoma allows for prevention by detecting and removing precursors before they undergo malignant transformation [3, 5], and screening remains the most powerful public health tool [6].

CRC screening has been implemented in several regions of China over the past decade, such as Guangzhou [7–9], Shanghai [10–12], Hangzhou [13] and Tianjin [14, 15]. Due to the limited economic and healthcare resources, a two-step sequential screening strategy was conducted in China [16–19], that a high-risk factors questionnaire (HRFQ) was parallel used with fecal immunochemical test (FIT) as preliminary screening to select high-risk participants for further colonoscopy confirmation. Nevertheless, accumulated data show the relatively low positive predictive values of the questionnaire plus FIT for selecting high-risk individuals [17, 20], as well as the low adherence to colonoscopy follow-up among those who should take [2, 11].

Previous studies assessing CRC screening program in China were mainly restricted to the performance of HRFQ and FIT, with other risk factors not analyzed [7–13]. Identifying risk factors for neoplasia is also essential for individualized screening to increase the cost-effectiveness of screening resources [21, 22]. Additionally, although a variety of potential risk factors for CRC have been identified, such as smoking, alcohol consumption, obesity, physical activity and dietary factors [23–30], they are based almost entirely on occurrence in older cohorts, and whether the findings are consistent across ages is unknown. Bailey et al. [31] predicted that in 2030, the incidence rate for colon and rectal cancer would increase by 27.7% and 46.0% for patients aged 35–49 years. However, to our knowledge, only a few studies have examined the characteristics, prevalence and related risk factors among young patients with colorectal neoplasia [32–36].

The objective of our study was to assess the neoplasia detection rates in high-risk populations, stratified by age and sex, while also identifying risk factors associated with colorectal neoplasia, therefore contributing to filling the existing knowledge gap.

## Material and methods

### Screening strategy and study population

This cross-sectional study was conducted under the framework of Tianjin CRC Screening Program, which was initiated in 2012 according to the *Technical Plan for Early Diagnosis and Early Treatment of Colorectal Cancer*formulated by the National Health Commission of the People’s Republic of China [15]. Briefly, residents aged 40–74 years were preliminarily screened by a HRFQ or FIT, and in the second step, those with either positive HRFQ or positive FIT were defined as high-risk participants and further recommended to undergo colonoscopy at a hospital designated by the program free of charge [9, 37]. All the screening-related testing was conducted in CRC screening units designated by Tianjin Health Commission.

**Fig. 1:**
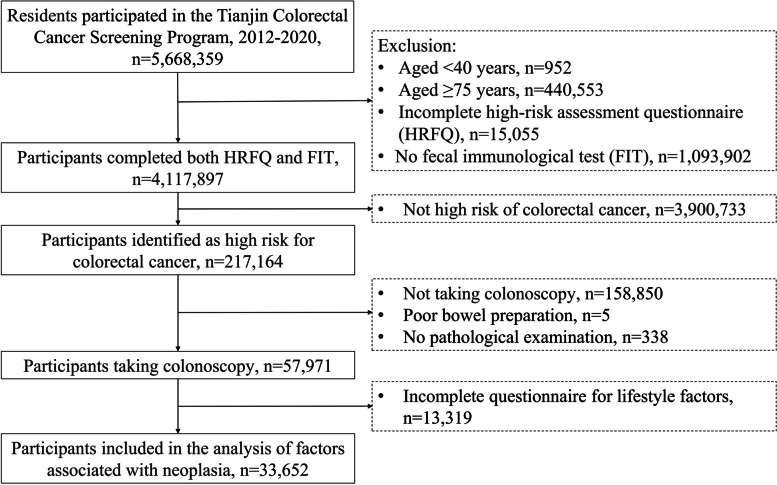
Flow diagram of participant recruitment in Tianjin Cancer Screening Program, 2012–2020. Abbreviations: CRC, colorectal cancer; HRFQ, high risk factor questionnaire; FIT, fecal immunological test

From 2012 to 2020, a total 5,668,359 community residents were recruited in the preliminary screening. After excluding participants aged < 40 or > 74 years (*n* = 441,505), those with invalid HRFQ (*n* = 15,055) and those with no FIT (*n* = 1,093,902), 4,117,897 participants were included in the analysis of preliminary screening (Fig. 1).

### High risk factor questionnaire

Participants who had one or more of the following risk factors are defined as HRFQ positive: (1) a family history of first-degree relatives (FDR) with CRC; (2) a personal history of any cancer; (3) a personal history of colorectal polyps; or (4) coexistence of at least two of the following syndromes: chronic diarrhoea; chronic constipation; mucus bloody stool; history of chronic appendicitis or appendectomy; history of chronic cholecystitis or cholecystectomy; history of psychiatric trauma (e.g., divorce, death of relatives) in the past 20 years.

### Fecal immunological tests

A fecal occult blood test was performed using the immunogold method (Abbott Biotechnology Co., Ltd.). With no diet restriction, participants were asked to collect a 10–50 mg stool sample and send it to a screening hospital laboratory within 8 h after collection. All tests were performed strictly according to the manufacturer's protocol. The FIT positivity threshold was set at 100 ng hemoglobin (Hb)/ml.

### Clinical procedures and definitions

All endoscopic examinations were performed by experienced endoscopists who had at least 5 years of experience and were all board certified to perform endoscopy. All abnormal findings were confirmed by expert gastrointestinal pathologists following up-to-date clinical guidelines.

We categorized colonoscopy findings into 3 groups: advanced neoplasia, nonadvanced neoplasia (equivalent to nonadvanced adenoma), and normal colonoscopy. Advanced neoplasia was defined as CRC or advanced adenoma ≥ 10 mm in diameter or with villous components or high-grade dysplasia. Normal colonoscopy referred to a colonoscopy in which no adenoma or CRC was found. Smoking status was categorized as never smoker, current smoker and former smoker. Alcohol intake status was categorized as never drinker and ever drinker, with the former including those never drinking and rarely drinking. Regular exercise was defined as more than 30 min of physical activity at least once per week; otherwise, it was classified as ‘irregular exercise’. For subsite, neoplasia in the cecum, ascending colon, hepatic flexure, transverse colon or splenic flexure were classified as proximal; neoplasia in the descending or sigmoid colon as distal, and those in the rectum or rectosigmoid junction as rectal.

### Ethics

The colorectal cancer screening protocol was approved by the Health Bureau of Tianjin City. This study was approved by the ethics committee of Tianjin Union Medicine Center. All participants signed written informed consent before information collection and colonoscopy examination. All investigations and methods used were in accordance with the Declaration of Helsinki.

### Statistical analysis

If a patient had multiple colonoscopies, only the first one recorded was included. Continuous variables were described as mean with standard deviation (SD), while categorical variables were described as frequency with percentage. Chi-square tests or Fisher’s exact test were used to compare categorical variables and one-way anova were used to compare continuous variables. Cochran-Armitage test for trend was performed to assess the association between detection rates and age groups. We conducted forward stepwise multivariable logistic regression analysis to identify potential risk factors for neoplasia; odds ratios (ORs) and 95% confidence intervals (CIs) were calculated. Forest plots were generated to visualize the results of multivariable logistic analysis. All analyses were performed using R software (V.4.1.2). Two-sided P values < 0.05 were considered to be statistically significant.

## Results

As shown in Fig. 1, a total of 4,117,897 eligible residents aged 40–74 years completed both a HRFQ and FIT in Tianjin CRC screening Program from 2012 to 2020. Of these, 143,661 (3.5%) were identified with a positive HRFQ, 88,459 (2.1%) with a positive FIT, and 217,164 (5.3%) were finally identified as high-risk participants. The characteristics of the overall and high-risk population are presented in Table 1. More females (53.2%) than males participated in screening. The mean age of overall participants was 60.80 ± 8.35 years; 95.4% were married and 10.0% had a college or above educational level. The proportions of participants with symptoms of chronic diarrhoea, chronic constipation and mucus bloody stool were 2.8%, 4.5% and 1.0% in the overall population and were 20.7%, 26.1% and 12.4% in the high-risk population.

**Table 1:** Characteristics of Participants in the Tianjin Colorectal Cancer Screening Program, 2012–2020

Characteristic	Total participants (*n* = 4,117,897)	High-risk participants (*n* = 217,164)	*P* value
**Age (years), mean (SD)**	60.80 (8.35)	63.51 (7.59)	< 0.001
40–44	74,832 (1.8)	1456 (0.7)	< 0.001
45–49	384,721 (9.3)	9521 (4.4)	
50–54	600,919 (14.6)	19,817 (9.1)	
55–59	732,385 (17.8)	33,446 (15.4)	
60–64	735,745 (17.9)	41,960 (19.3)	
65–69	866,164 (21.0)	56,502 (26.0)	
70–74	723,131 (17.6)	54,462 (25.1)	
**Sex, n (%)**			
Male	1,927,100 (46.8)	89,371 (41.2)	< 0.001
Female	2,190,797 (53.2)	127,793 (58.8)	
**Marital status, n (%)**			
Married	3,929,779 (95.4)	181,399 (83.5)	< 0.001
Unmarried	47,734 (1.2)	1826 (0.8)	
Divorced	29,129 (0.7)	5740 (2.6)	
Widowed	91,535 (2.2)	27,966 (12.9)	
Unknown	19,720 (0.5)	233 (0.1)	
**Educational level, n (%)**			
Primary school or below	1,046,679 (25.4)	62,378 (28.7)	< 0.001
Middle school	2,615,698 (63.5)	134,673 (62.0)	
College or above	411,378 (10.0)	19,454 (9.0)	
Unknown	44,142 (1.1)	659 (0.3)	
**Factors for risk stratification, n (%)**			
Chronic diarrhoea	116,248 (2.8)	44,888 (20.7)	< 0.001
Chronic constipation	185,216 (4.5)	56,691 (26.1)	< 0.001
Mucus bloody stool	40,839 (1.0)	26,900 (12.4)	< 0.001
Chronic appendicitis/appendectomy	76,864 (1.9)	27,485 (12.7)	< 0.001
Chronic cholecystitis/cholecystectomy	61,156 (1.5)	24,808 (11.4)	< 0.001
Psychiatric trauma	132,984 (3.2)	48,719 (22.4)	< 0.001
Personal history of any cancer	12,192 (0.3)	12,192 (5.6)	< 0.001
Personal history of colorectal polyps	11,618 (0.3)	11,618 (5.3)	< 0.001
Family history of CRC in FDR	21,415 (0.5)	21,415 (9.9)	< 0.001
FIT positive	88,459 (2.1)	88,459 (40.7)	< 0.001

*Abbreviations*: *CRC* Colorectal cancer, *FIT* Fecal immunological test, *FDR* First-degree relative

### Preliminarily screening

**Fig. 2:**
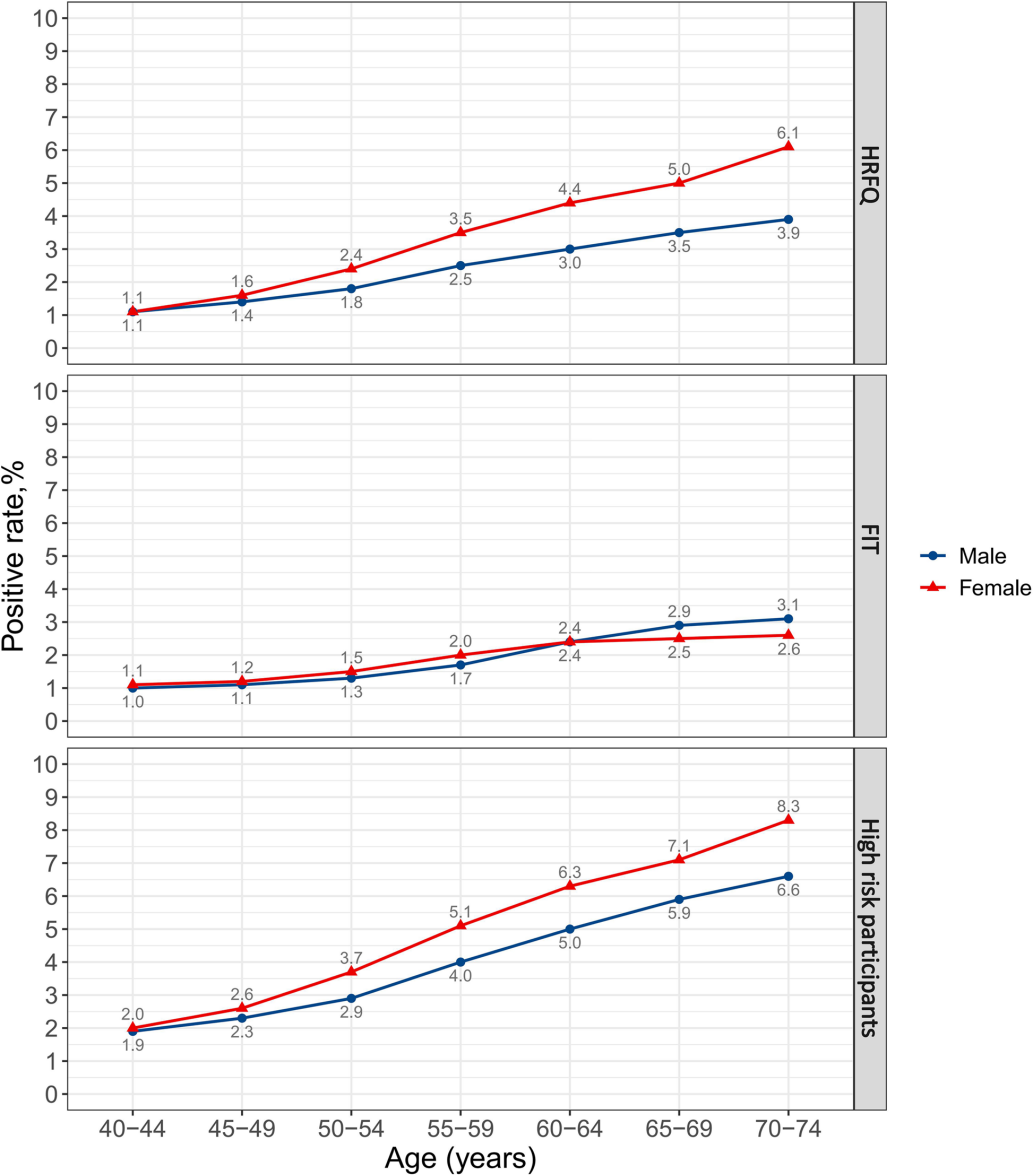
Positive rates of HRFQ, FIT and preliminary screening by age and sex. Abbreviations: HRFQ, high risk factor questionnaire; FIT, fecal immunological test

The positive rates of HRFQ, FIT and preliminarily screening strongly increased with age (Table 2; Fig. 2). For individuals aged 40–44, 45–49, 50–54, 55–59, 60–64, 65–69 and 70–74 years, the positive rates of preliminarily screening were 1.9%, 2.5%, 3.3%, 4.6%, 5.7%, 6.5% and 7.5%, respectively (*P* for trend < 0.001). Females had significantly higher positive rates of HRFQ and preliminarily screening than males in the overall population (4.1% vs. 2.8%, *p* < 0.001; 5.8% vs. 4.6%, *P* < 0.001, respectively) and in age groups older than 50 (all *P* < 0.001). For FIT, the overall positive rate was higher in males (2.2% vs. 2.1%, *P* = 0.002), although an opposite trend was observed in those aged < 60 years (Fig. 2).

**Table 2:** The Results of Preliminary Screening and Colonoscopy Examination by Age Group

	**Participants for preliminary screening**				**Participants undertaking colonoscopy examination**			
	**Total participants**	**HRFQ positive (%)**	**FIT positive (%)**	**High-risk participants (%)**	**Total number**	**Cases of nonadvanced adenoma (%)**	**Cases of advanced adenoma (%)**	**Cases of CRC (%)**
40–44	74,832	839 (1.1)	765 (1.0)	1456 (1.9)	467	116 (24.8)	11 (2.4)	2 (0.4)
45–49	384,721	5830 (1.5)	4442 (1.2)	9521 (2.5)	2393	628 (26.2)	43 (1.8)	5 (0.2)
50–54	600,919	12,758 (2.1)	8504 (1.4)	19,817 (3.3)	4793	1524 (31.8)	163 (3.4)	22 (0.5)
55–59	732,385	22,252 (3.0)	13,586 (1.9)	33,446 (4.6)	9007	3244 (36.0)	357 (4.0)	79 (0.9)
60–64	735,745	27,682 (3.8)	17,426 (2.4)	41,960 (5.7)	12,095	4626 (38.2)	686 (5.7)	143 (1.2)
65–69	866,164	37,269 (4.3)	23,042 (2.7)	56,502 (6.5)	15,727	6308 (40.1)	958 (6.1)	290 (1.8)
70–75	723,131	37,031 (5.1)	20,694 (2.9)	54,462 (7.5)	13,489	5382 (39.9)	991 (7.3)	322 (2.4)
Overall	4,117,897	143,661 (3.5)	88,459 (2.1)	217,164 (5.3)	57,971	21,828 (37.7)	3209 (5.5)	863 (1.5)
P for trend		< 0.001	< 0.001	< 0.001		< 0.001	< 0.001	< 0.001

*Abbreviations*: *HRFQ* High risk factor questionnaire, *FIT* Fecal immunological test, *CRC* Colorectal cancer

^a^ *P* value was calculated by the Cochran-Armitage test for trend

### Colorectal neoplasia detection rates

**Fig. 3:**
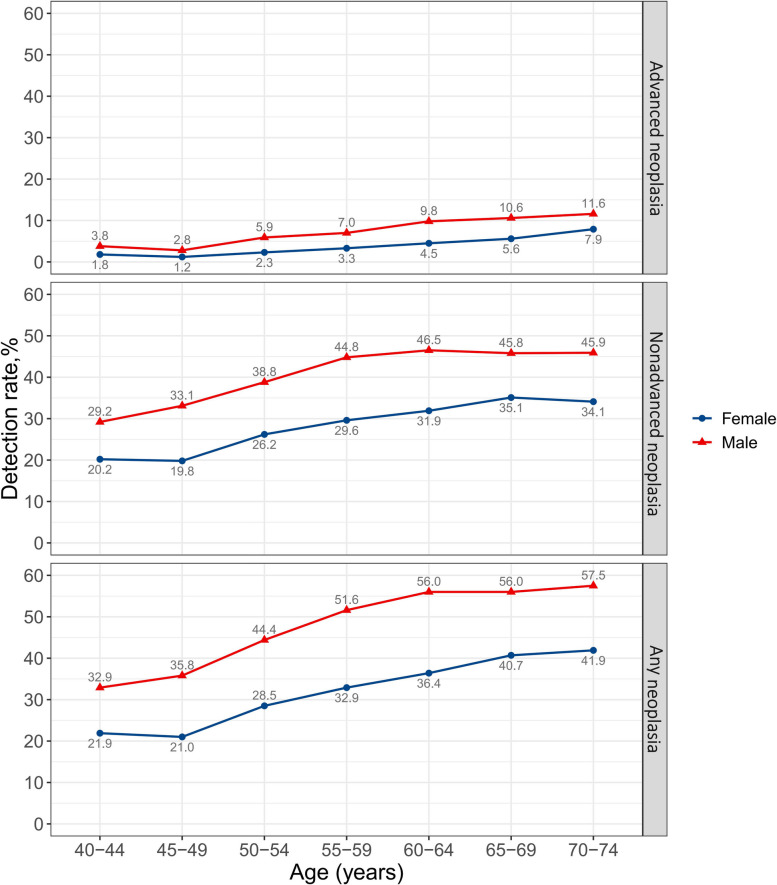
Detection rates of advanced neoplasia, nonadvanced neoplasia and any neoplasia stratified by age and sex

In the second stage of CRC screening, 57,971 preliminary positive participants subsequently undertook a screening colonoscopy following the screening physicians’ recommendations. Of these, 21,828 (37.7%), 3209 (5.5%) and 863 (1.5%) were diagnosed as nonadvanced adenoma, advanced adenoma and CRC, respectively (Table 2). As shown in Fig. 3, the detection rates of colorectal neoplasia increased after the age of 50, and were substantially higher in males than in females across all age groups (all P < 0.001). In males, the detection rate of nonadvanced neoplasia increased from 40 to 60 years of age and remained approximately constant after the age of 60, while in females, it showed no increase before the age of 50 but continuously increased up to 74 years of age. No increase was observed in the detection rate of advanced neoplasia among those younger than 50 in both sexes.

### Factors associated with the colorectal neoplasia detection

To identify potential risk factors that may be associated with colorectal neoplasia, we exclude 13,319 participants with incomplete questionnaire for lifestyle factors, and 33,652 remaining participants were included in the following analysis, with the characteristics shown in Table 3. The mean age was 62.89 ± 7.13 years; 53.4% were females, 19.3% current smoker, 10.9% ever drinker, 44.5% exercised regularly and 43.6% had a higher BMI (≥ 25 kg/m [2]). Compared with participants who did not develop neoplasia, those with advanced neoplasia or nonadvanced adenoma were more likely to smoke, and drink alcohol, exercise regularly and have a higher BMI.

**Table 3:** Characteristics of the Study Population

Characteristic	Overall	Normal	Nonadvanced neoplasia	Advanced neoplasia	*P*
	(***n*** = 33,652)	(***n*** = 16,827)	(***n*** = 14,194)	(***n*** = 2631)	
**Age (years), mean (SD)**	62.89 ± 7.13	61.96 ± 7.45	63.55 ± 6.71	65.25 ± 6.16	< 0.001
40–44	375 (1.1)	275 (1.6)	91 (0.6)	9 (0.3)	< 0.001
45–49	1218 (3.6)	835 (5.0)	356 (2.5)	27 (1.0)	
50–54	2818 (8.4)	1716 (10.2)	990 (7.0)	112 (4.3)	
55–59	5732 (17.0)	3124 (18.6)	2306 (16.2)	302 (11.5)	
60–64	7885 (23.4)	3839 (22.8)	3420 (24.1)	626 (23.8)	
65–69	9387 (27.9)	4320 (25.7)	4209 (29.7)	858 (32.6)	
70–74	6237 (18.5)	2718 (16.2)	2822 (19.9)	697 (26.5)	
**Sex, n (%)**					
Male	15,682 (46.6)	6350 (37.7)	7707 (54.3)	1625 (61.8)	< 0.001
Female	17,970 (53.4)	10,477 (62.3)	6487 (45.7)	1006 (38.2)	
**Marital status, n (%)**					
Married	32,151 (95.5)	16,047 (95.4)	13,587 (95.7)	2517 (95.7)	0.500
Unmarried	299 (0.9)	160 (1.0)	114 (0.8)	25 (1.0)	
Divorced	270 (0.8)	150 (0.9)	100 (0.7)	20 (0.8)	
Widowed	857 (2.5)	427 (2.5)	366 (2.6)	64 (2.4)	
Unknown	75 (0.2)	43 (0.3)	27 (0.2)	5 (0.2)	
**Educational level, n (%)**					
Primary school or below	7053 (21.0)	3594 (21.4)	2955 (20.8)	504 (19.2)	0.079
Middle school	22,359 (66.4)	11,106 (66.0)	9458 (66.6)	1795 (68.2)	
College or above	4084 (12.1)	2037 (12.1)	1727 (12.2)	320 (12.2)	
Unknown	156 (0.5)	90 (0.5)	54 (0.4)	12 (0.5)	
**Smoking status, n (%)**					
Never smoker	25,548 (75.9)	13,731 (81.6)	10,078 (71.0)	1739 (66.1)	< 0.001
Former smoker	1608 (4.8)	655 (3.9)	790 (5.6)	163 (6.2)	
Current smoker	6496 (19.3)	2441 (14.5)	3326 (23.4)	729 (27.7)	
**Alcohol drinking, n (%)**					
Never drinker	29,996 (89.1)	15,533 (92.3)	12,280 (86.5)	2183 (83.0)	< 0.001
Ever drinker	3656 (10.9)	1294 (7.7)	1914 (13.5)	448 (17.0)	
**Physical activity, n (%)**					
Irregular exercise	18,673 (55.5)	9660 (57.4)	7668 (54.0)	1345 (51.1)	< 0.001
Regular exercise	14,979 (44.5)	7167 (42.6)	6526 (46.0)	1286 (48.9)	
**BMI, n (%)**					
< 25	18,953 (56.3)	10,023 (59.6)	7581 (53.4)	1349 (51.3)	< 0.001
25–29.9	12,502 (37.2)	5855 (34.8)	5561 (39.2)	1086 (41.3)	
≥ 30	2197 (6.5)	949 (5.6)	1052 (7.4)	196 (7.4)	
**Factors for risk stratification, n (%)**					
Chronic diarrhoea	7936 (23.6)	4300 (25.6)	3159 (22.3)	477 (18.1)	< 0.001
Chronic constipation	7967 (23.7)	4364 (25.9)	3154 (22.2)	449 (17.1)	< 0.001
Mucus or bloody stool	5896 (17.5)	3194 (19.0)	2310 (16.3)	392 (14.9)	< 0.001
Chronic appendicitis/appendectomy	3025 (9.0)	1568 (9.3)	1286 (9.1)	171 (6.5)	< 0.001
Chronic cholecystitis/cholecystectomy	2889 (8.6)	1510 (9.0)	1186 (8.4)	193 (7.3)	0.009
Psychiatric trauma	2941 (8.7)	1594 (9.5)	1191 (8.4)	156 (5.9)	< 0.001
Personal history of any cancer	1388 (4.1)	713 (4.2)	514 (3.6)	161 (6.1)	< 0.001
Personal history of colorectal polyps	3504 (10.4)	1271 (7.6)	1978 (13.9)	255 (9.7)	< 0.001
Family history of CRC in first degree relatives	3584 (10.7)	1718 (10.2)	1603 (11.3)	263 (10.0)	0.011
FIT positive	20,310 (60.4)	9944 (59.1)	8497 (59.9)	1869 (71.0)	< 0.001

*Abbreviations*: *BMI* Body Mass Index, *CRC* Colorectal cancer, *FIT* Fecal immunological test, *SD* Standard deviation

**Fig. 4:**
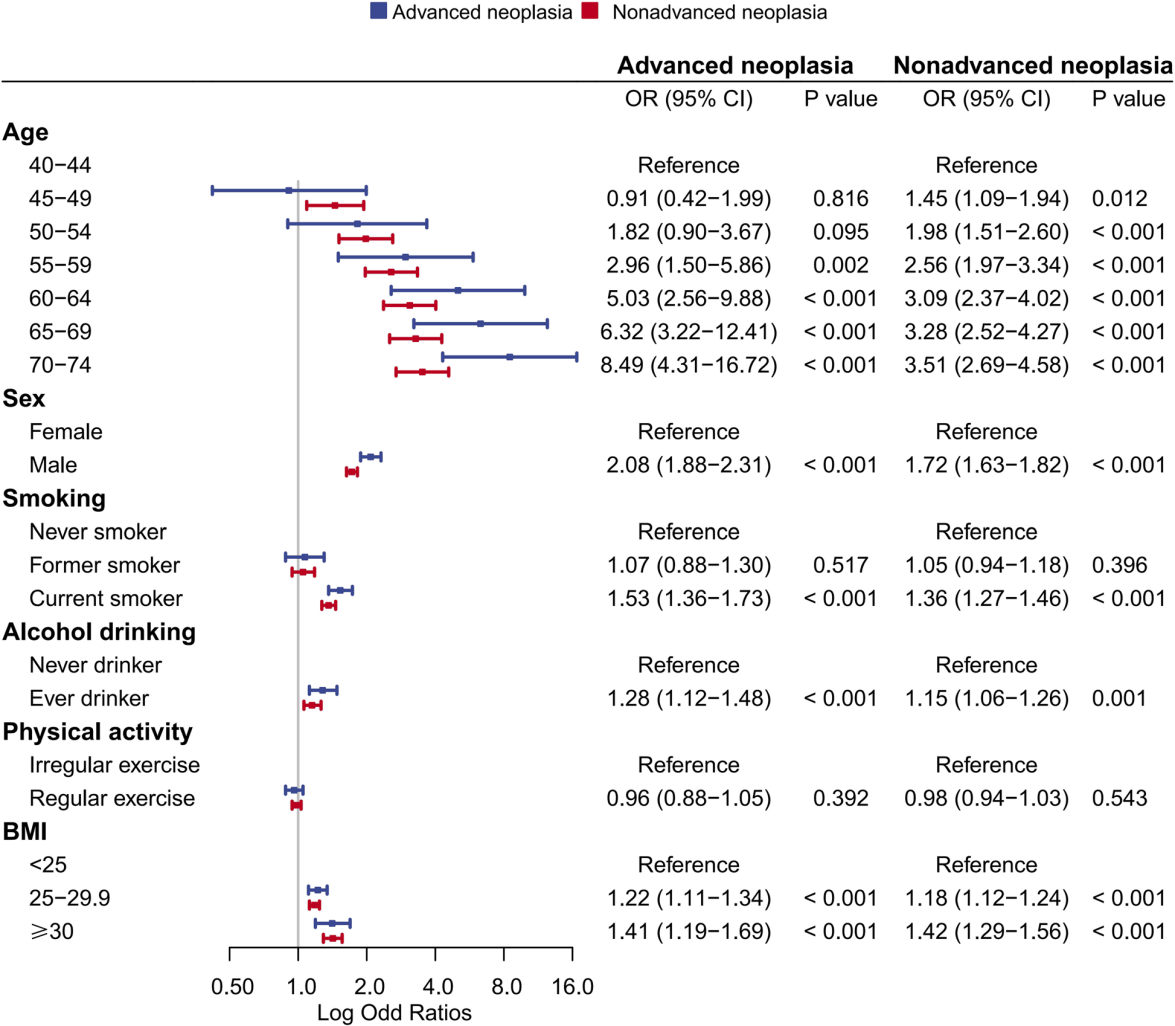
Multivariable analyses on the risk for advanced neoplasia and nonadvanced adenoma. Analyses were adjusted for sex, educational level, marital status, year of colonoscopy, smoking status, alcohol drinking, physical activity, body mass index, chronic diarrhoea, chronic constipation, mucus bloody stool, chronic appendicitis or appendectomy, chronic cholecystitis or cholecystectomy, psychiatric trauma in the past 20 years, personal history of any cancer, personal history of colorectal polyps and family history of colorectal cancer in first degree relatives. Abbreviations: OR, Odds ratio; CI, confidence interval; BMI, body mass index

As shown in Fig. 4, older age, male sex, current smoker, ever drinker, and a BMI ≥ 25 kg/m [2] were significantly associated with increased odds for both advanced and nonadvanced neoplasia. Older age was the most powerful predictor for neoplasia: compared with individuals aged 40–44 years, the adjusted ORs (95%CI) of individuals aged 45–49, 50–54, 55–59, 60–64 65–69 and 70–74 years were 01.45, 1.98, 2.56, 3.09, 3.28 and 3.51, respectively for nonadvanced neoplasia and were 0.91, 1.82, 2.96, 5.03, 6.32 and 8.49, respectively for advanced neoplasia. No association was observed for regular exercise with neoplasia in our study population.

### Factors associated with the colorectal neoplasia detection by age groups

Table 4 presents the results of multivariable logistic regression analyses for advanced and nonadvanced neoplasia by age groups, while analyses stratified by sex are provided in Supplementary Table 1 and Supplementary Table 2. Male sex was the only independent risk factor in the 40–49-year-old group. And the association appeared to be stronger in younger groups than in older groups. For instance, male sex had a 2.50-fold increase in risk of advanced neoplasia in the 40–49-year group, which was 2.24-, and 2.09-fold in those aged 50–59 and 60–74 years, respectively. In the 50–59-year group, male sex, current smoking and drinking were positively significantly associated with the presence of both advanced and nonadvanced neoplasia. Risk factors in the 60–74-year group were similar to those in the 60–69-year group, and higher BMI was an additional risk factor in the 60–74-year group. Overall, the associations for advanced neoplasia were stronger than those for nonadvanced neoplasia. No association was found between being a former smoker and higher risk, irrespective of age and lesions. Regular exercise was also not associated with either advanced or nonadvanced neoplasia in our population.

**Table 4:** Multivariable Analyses on the Risk for Advanced Neoplasia and Nonadvanced Adenoma Stratified by Age Groups

	**40–49 years**		**50–59 years**		**60–74 years**	
**Variable**	**Adjusted OR**^**a**^	***P***	**Adjusted OR**^**a**^	***P***	**Adjusted OR**^**a**^	***P***
	**(95% CI)**		**(95% CI)**		**(95% CI)**	
**For advanced neoplasia**						
Sex (vs. female)						
Male	2.50 (1.05–5.98)	0.039	2.24 (1.72–2.92)	< 0.001	2.09 (1.86–2.34)	< 0.001
Smoking status (vs. never)						
Former	NA^b^	0.991	1.13 (0.62–2.07)	0.686	1.09 (0.89–1.34)	0.407
Current	1.53 (0.60–3.95)	0.375	1.48 (1.09–2.02)	0.012	1.51 (1.32–1.72)	< 0.001
Alcohol drinking (vs. never)						
Ever drinker	0.80 (0.20–3.15)	0.753	1.81 (1.28–2.57)	< 0.001	1.23 (1.05–1.43)	0.009
Physical activity (vs. irregular exercise)						
Regular exercise	0.87 (0.33–2.29)	0.777	1.07 (0.84–1.37)	0.580	1.01 (0.92–1.12)	0.806
BMI, kg/m2 (vs. < 25)						
25–29.9	1.03 (0.46–2.26)	0.950	1.11 (0.88–1.40)	0.386	1.26 (1.14–1.40)	< 0.001
≥ 30	1.44 (0.31–6.76)	0.645	1.35 (0.85–2.15)	0.209	1.47 (1.22–1.78)	< 0.001
**For nonadvanced neoplasia**						
Sex (vs. female)						
Male	1.92 (1.46–2.52)	< 0.001	1.91 (1.71–2.14)	< 0.001	1.66 (1.55–1.77)	< 0.001
Smoking status (vs. never)						
Former	0.62 (0.16–2.32)	0.477	0.81 (0.60–1.10)	0.181	1.13 (1.00–1.28)	0.058
Current	1.21 (0.86–1.69)	0.275	1.27 (1.10–1.46)	0.001	1.40 (1.29–1.52)	< 0.001
Alcohol drinking (vs. never)						
Ever drinker	1.15 (0.73–1.81)	0.536	1.22 (1.01–1.47)	0.040	1.14 (1.03–1.26)	0.010
Physical activity (vs. irregular exercise)						
Regular exercise	1.22 (0.91–1.65)	0.185	1.05 (0.94–1.17)	0.374	0.98 (0.93–1.04)	0.543
BMI, kg/m2 (vs. < 25)						
25–29.9	1.20 (0.93–1.56)	0.160	1.08 (0.98–1.20)	0.117	1.22 (1.15–1.30)	< 0.001
≥ 30	1.40 (0.79–2.48)	0.250	1.23 (0.99–1.52)	0.058	1.49 (1.33–1.67)	< 0.001

*Abbreviations*: *OR* Odds ratio, *CI* Confidence interval, *BMI* Body mass index

^a^ ORs were adjusted for sex, educational level, marital status, year of colonoscopy, smoking status, alcohol drinking, physical activity, body mass index, chronic diarrhoea, chronic constipation, mucus or bloody stool, chronic appendicitis/appendectomy, chronic cholecystitis/cholecystectomy, psychiatric trauma in the past 20 years, personal history of any cancer, personal history of colorectal polyps and family history of colorectal cancer in first degree relatives; ^b^Not applicable owing to only 1 advanced neoplasia in this subgroup

## Discussion

In this study, we reported the results of Tianjin CRC screening program from 2012 to 2020. For 4,117,897 participants completed both a HRFQ and FIT, the positive rates of HRFQ, FIT and preliminary screening were 3.5%, 2.1% and 5.3%, respectively and all increased with age. For 57,971 participants undertaking colonoscopy, the detection rates of nonadvanced adenoma, advanced adenoma and CRC were 37.8%, 5.7% and 1.6%, respectively, and pronounced increases was observed from the age of 50. Females had higher positive rate of preliminary screening while males had higher neoplasia detection rate. Older age, male sex, current smoker, ever drinker, and higher BMI were significantly associated with the presence of both advanced and nonadvanced neoplasia. Whereas, we found that associations of risk factors with increased neoplasia risk were mainly restricted to individuals aged above 50 but not those aged 40–49 years, which may be helpful in designing colonoscopy-based screening programs and optimization.

In present study, the detection rate of CRC was 1.5%, which was comparable to 1.2% in Jiashan [13], 1.17%-1.6% in Guangzhou [7–9], but was lower than 2.3% in Shanghai [12] and higher than 1.2% from a rural population in Zhejiang [17]. Additionally, the detection rate of any colorectal neoplasia was 44.7% in our population, higher than 16.3%-43.5% from other cities [8, 9, 13, 17]. These results mean that preliminary positive participants would have a 1.5% possibility of having CRC and a 44.7% possibility of having any colorectal neoplasia. However, there were 55.4% participants who were positive in the preliminary screening and underwent colonoscopies but with no colorectal neoplasia detected. Further efforts to enable more targeted offers for colonoscopy in screening are highly required.

Our study revealed that the positivity rate of HRFQ in all participants was 3.5%, which was higher than the positivity rate of FIT at 2.1%. This trend was consistent across various age groups, mirroring the findings of a study conducted in Guangzhou, China [38]. While the positivity rate of HRFQ is relatively high, the results indicated that HRFQ has a lower detection rate for advanced adenoma and CRC compared to FIT. Previous research also supports that FIT remains the most significant non-invasive screening tool for colorectal cancer [39]. HRFQ lags behind FIT in the screening of CRC and advanced adenoma, but excels in the screening of nonadvanced adenoma when compared to FIT, making it a valuable complementary approach to FIT. Meng et al. [40] and Wong et al. [41] have reported that FIT demonstrates a better detection rate for CRC and advanced adenoma, especially in cases associated with bleeding. However, for non-bleeding nonadvanced adenoma and advanced adenoma cases, HRFQ can serve as a robust supplement to FIT. HRFQ encompasses multiple colorectal tumor risk indicators and is user-friendly. Combining HRFQ with FIT in China as the initial step in colorectal cancer screening significantly enhances the efficiency of the initial screening process.

Although the incidence of CRC patients aged 50 years and above has been reduced over the past few decades, there appears an opposite trend among younger patients in a substantial number of countries [41–44]. The latest recommendations from the US Preventive Services Task Force lowered the age for initiating average-risk CRC screening from 50 to 45 years [45]. In our population, no increase was observed in detection of advanced neoplasia among those younger than 50, but we found a pronounced increase in detection of nonadvanced adenoma in males aged 40–49 years. In addition, we found that male sex, identified as an independent risk factor, had an even greater impact on young individuals aged 40–49 than on older individuals. To date, guidelines have been consistent in suggesting the same age of initiation for screening for males and females. Our findings support that the age at which to start screening should be sex specific.

Our study shows that several lifestyle factors including smoking, drinking and BMI were associated with the presence of colorectal neoplasia among individuals aged above 50 but not those aged 40–49 years. Risk factors for colorectal neoplasia have been extensively explored but they were mostly obtained from a survey of older cohort [45–49]. Limited studies have examined risk factors for early colorectal neoplasia (age under 50 years). A study from Italy found no apparent risk factors for colorectal neoplasia in patients aged 40–49 [36]. Another study of United States veterans 18–49 years old showed that current smoking was not significantly associated with increased risk and increased BMI was even a protective factor for early-onset CRC [33]. A possible explanation is the relatively short exposure of young adults, such as the relationship between increased risk and more years of smoking [50]. The inconsistency also could be partly due to the distinctive biologic phenotype of early-onset CRC, which is very different from that in older patients [52, 53].

Two neoplasia outcomes were considered in the present study: advanced and nonadvanced neoplasia. With respect to potential implications for the design of CRC screening programs, advanced neoplasia is the most relevant outcome. However, it would be more reassuring and convincing if the effect of risk factors was consistent for nonadvanced and advanced neoplasia because both are considered to be part of the multistage model of colorectal carcinogenesis [54].

Similar to most countries, high-risk assessment in China is mainly based on family history of CRC, personal history of polyps and some gastrointestinal symptoms but does not take lifestyle factors into account. However, it has been suggested that men with abdominal obesity or metabolic syndrome might benefit from colonoscopy screening starting at 45 years of age [35]. A cost-effectiveness analysis suggested that increasing participation rates among unscreened older individuals at higher risk would be more cost effective than initiating screening at age 45 years [55]. Given the relatively low positive predict value of preliminary screening and low participation rate in colonoscopy in China, elucidating age-specific risk factors is essential for individualized screening to increase the cost-effectiveness, which may serve to clarify which individuals at different ages should be prioritized and which risk factors might be helpful in the design of colonoscopy-based screening programs.

This study had some limitations. First, the cross-sectional nature of the study limits the causality interpretation. Second, the exposures were based on self-reports and might include some misclassification. Third, the data were collected in Tianjin, China, and were not applicable to represent the whole country. Fourth, some other potential factors, such as the intake of fruit, vegetable and red and processed meat, were not included, and the type of alcohol (beer, wine, distilled spirit) was not taken into account, which might influence the accuracy of the results.

## Conclusions

In summary, we report the results of Tianjin CRC screening program from 2012 to 2020 and further identified risk factors of colorectal neoplasia. We found an earlier increase in detection of neoplasia in males than in females, suggesting that initiating age for screening should be sex specific. Further, we extend prior studies by demonstrating that the associations of smoking, drinking and BMI with neoplasia detection are mainly restricted to individuals aged above 50 years, which may serve to clarify which individuals at different ages should be prioritized and which risk factors might be helpful in the design of colonoscopy-based screening programs. Above all, our findings highlight that age-specific risk stratification and sex-specific initiating ages for CRC screening might be recommended to improve the accuracy and effectiveness of current screening strategy.

### Supplementary Information

**Additional file 1:** **Table 1.** Multivariable Analyses on the Risk for Advanced Neoplasia and Nonadvanced Adenoma Stratified in Male Sex. **Table 2.** Multivariable Analyses on the Risk for Advanced Neoplasia and Nonadvanced Adenoma Stratified in Female Sex.

## Data Availability

The datasets generated and analyzed during the current study are available from the corresponding author on reasonable request.

## Abbreviations

CRC: Colorectal cancer
FIT: Faecal immunochemical test
HRFQ: High-risk factors questionnaire
BMI: Body mass index
FDR: First-degree relatives
Hb: Hemoglobin
SD: Standard deviation
OR: Odds ratio
CI: Confidence interval
