# Supplementary material for: Age- and sex-stratified detection rates and associated factors of colorectal neoplasia in the Tianjin colorectal cancer screening program from 2012 to 2020
Source: BMC Gastroenterol. 2023 Dec 12;23:435. doi: 10.1186/s12876-023-03060-3 (PMC10714643; doi:10.1186/s12876-023-03060-3)
Supplement: Supplementary file 1 — Additional file 1: Table 1. Multivariable Analyses on the Risk for Advanced Neoplasia and Nonadvanced Adenoma Stratified in Male Sex. Table 2. Multivariable Analyses on the Risk for Advanced Neoplasia and Nonadvanced Adenoma Stratified in Female Sex. [file 12876_2023_3060_MOESM1_ESM.docx]

**Supplementary Materials**

**Supplementary Table 1.** Multivariable Analyses on the Risk for Advanced Neoplasia and Nonadvanced Adenoma Stratified in Male Sex

|  | **40-49 years** |  | **50-59 years** |  | **60-74 years** |  |
| --- | --- | --- | --- | --- | --- | --- |
| **Variable** | **Adjusted OR^a^** | **P** | **Adjusted OR^a^** | **P** | **Adjusted OR^a^** | **P** |
|  | **(95% CI)** |  | **(95% CI)** |  | **(95% CI)** |  |
| **For advanced neoplasia** | | | | | | |
| Smoking status (vs. never) |  |  |  |  |  |  |
| Former | NA^b^ | 0.986 | 1.25 (0.69-2.26) | 0.459 | 0.96 (0.77-1.19) | 0.688 |
| Current | 1.23 (0.50-3.04) | 0.648 | 1.25 (0.92-1.70) | 0.148 | 1.41 (1.22-1.62) | < 0.001 |
| Alcohol drinking (vs. never) |  |  |  |  |  |  |
| Ever drinker | 0.78 (0.21-2.91) | 0.709 | 1.62 (1.16-2.26) | 0.005 | 1.25 (1.07-1.45) | 0.004 |
| Physical activity (vs. irregular exercise) | | | | | | |
| Regular exercise | 1.03 (0.38-2.84) | 0.951 | 1.06 (0.79-1.43) | 0.693 | 1.03 (0.91-1.17) | 0.614 |
| BMI, kg/m2 (vs. <25) |  |  |  |  |  |  |
| 25-29.9 | 1.08 (0.46-2.55) | 0.860 | 1.22 (0.92-1.61) | 0.163 | 1.22 (1.07-1.39) | 0.002 |
| ≥30 | 1.92 (0.41-9.07) | 0.409 | 1.15 (0.62-2.14) | 0.660 | 1.36 (1.04-1.78) | 0.026 |
| **For nonadvanced neoplasia** | | | | | | |
| Smoking status (vs. never) |  |  |  |  |  |  |
| Former | 0.73 (0.20-2.68) | 0.631 | 1.18 (0.88-1.57) | 0.263 | 1.41 (1.25-1.59) | < 0.001 |
| Current | 1.56 (1.14-2.12) | 0.005 | 1.75 (1.54-1.99) | < 0.001 | 1.65 (1.53-1.78) | < 0.001 |
| Alcohol drinking (vs. never) |  |  |  |  |  |  |
| Ever drinker | 1.27 (0.82-1.96) | 0.277 | 1.42 (1.18-1.69) | < 0.001 | 1.36 (1.24-1.50) | < 0.001 |
| Physical activity (vs. irregular exercise) | | | | | | |
| Regular exercise | 1.21 (0.92-1.6) | 0.174 | 1.08 (0.98-1.19) | 0.130 | 1.01 (0.96-1.07) | 0.719 |
| BMI, kg/m2 (vs. <25) |  |  |  |  |  |  |
| 25-29.9 | 1.22 (0.96-1.56) | 0.102 | 1.08 (0.98-1.19) | 0.128 | 1.23 (1.16-1.30) | < 0.001 |
| ≥30 | 1.48 (0.86-2.55) | 0.155 | 1.22 (1.00-1.50) | 0.052 | 1.43 (1.28-1.60) | < 0.001 |

**Notes:** ^a^ORs were adjusted for sex, educational level, marital status, year of colonoscopy, smoking status, alcohol drinking, physical activity, body mass index, chronic diarrhoea, chronic constipation, mucus or bloody stool, chronic appendicitis/appendectomy, chronic cholecystitis/cholecystectomy, psychiatric trauma in the past 20 years, personal history of any cancer, personal history of colorectal polyps and family history of colorectal cancer in first degree relatives; ^b^Not applicable owing to only 1 advanced neoplasia in this subgroup.

**Abbreviations**: OR, Odds ratio; CI, confidence interval; BMI, body mass index.

**Supplementary Table 2.** Multivariable Analyses on the Risk for Advanced Neoplasia and Nonadvanced Adenoma Stratified in Female Sex

|  | **40-49 years** |  | **50-59 years** |  | **60-74 years** |  |
| --- | --- | --- | --- | --- | --- | --- |
| **Variable** | **Adjusted OR^a^** | **P** | **Adjusted OR^a^** | **P** | **Adjusted OR^a^** | **P** |
|  | **(95% CI)** |  | **(95% CI)** |  | **(95% CI)** |  |
| **For advanced neoplasia** | | | | | | |
| Smoking status (vs. never) |  |  |  |  |  |  |
| Former | NA^b^ | 0.986 | 1.77 (1.03-3.07) | 0.040 | 1.45 (1.19-1.76) | < 0.001 |
| Current | 1.82 (0.77-4.30) | 0.172 | 2.03 (1.55-2.66) | < 0.001 | 1.96 (1.74-2.21) | < 0.001 |
| Alcohol drinking (vs. never) |  |  |  |  |  |  |
| Ever drinker | 0.87 (0.23-3.31) | 0.839 | 1.91 (1.37-2.67) | < 0.001 | 1.57 (1.35-1.81) | < 0.001 |
| Physical activity (vs. irregular exercise) | | | | | | |
| Regular exercise | 1.14 (0.49-2.65) | 0.763 | 1.03 (0.82-1.29) | 0.803 | 1.06 (0.96-1.16) | 0.231 |
| BMI, kg/m2 (vs. <25) |  |  |  |  |  |  |
| 25-29.9 | 1.45 (0.72-2.95) | 0.300 | 1.21 (0.98-1.50) | 0.081 | 1.29 (1.17-1.42) | < 0.001 |
| ≥30 | 1.72 (0.38-7.67) | 0.480 | 1.25 (0.80-1.95) | 0.325 | 1.42 (1.18-1.71) | < 0.001 |
| **For nonadvanced neoplasia** | | | | | | |
| Smoking status (vs. never) |  |  |  |  |  |  |
| Former | 0.61 (0.16-2.30) | 0.464 | 0.94 (0.68-1.3) | 0.716 | 1.08 (0.94-1.23) | 0.296 |
| Current | 1.15 (0.82-1.61) | 0.416 | 1.18 (1.01-1.37) | 0.039 | 1.26 (1.15-1.39) | < 0.001 |
| Alcohol drinking (vs. never) |  |  |  |  |  |  |
| Ever drinker | 1.2 (0.77-1.86) | 0.421 | 1.19 (0.99-1.43) | 0.071 | 1.14 (1.03-1.26) | 0.010 |
| Physical activity (vs. irregular exercise) | | | | | | |
| Regular exercise | 0.87 (0.60-1.27) | 0.482 | 1.09 (0.94-1.27) | 0.240 | 1.00 (0.92-1.07) | 0.543 |
| BMI, kg/m2 (vs. <25) |  |  |  |  |  |  |
| 25-29.9 | 1.05 (0.77-1.43) | 0.768 | 1.01 (0.88-1.17) | 0.868 | 1.21 (1.12-1.32) | < 0.001 |
| ≥30 | 1.59 (0.84-3.03) | 0.157 | 1.21 (0.89-1.66) | 0.232 | 1.57 (1.32-1.87) | < 0.001 |

**Notes:** ^a^ORs were adjusted for sex, educational level, marital status, year of colonoscopy, smoking status, alcohol drinking, physical activity, body mass index, chronic diarrhoea, chronic constipation, mucus or bloody stool, chronic appendicitis/appendectomy, chronic cholecystitis/cholecystectomy, psychiatric trauma in the past 20 years, personal history of any cancer, personal history of colorectal polyps and family history of colorectal cancer in first degree relatives; ^b^Not applicable owing to only 1 advanced neoplasia in this subgroup.

**Abbreviations**: OR, Odds ratio; CI, confidence interval; BMI, body mass index.
